# Assessing the effectiveness of ‘pulse radiofrequency treatment of dorsal root ganglion’ in patients with chronic lumbar radicular pain: study protocol for a randomized control trial

**DOI:** 10.1186/1745-6215-13-52

**Published:** 2012-04-28

**Authors:** Harsha Shanthanna, Philip Chan, James McChesney, James Paul, Lehana Thabane

**Affiliations:** 1 Department of Anesthesia and Pain Medicine (D-149), St Joseph’s Hospital, 50 Charlton Avenue East Hamilton, Ontario, L8N 4A6, Canada; 2 Department of Anesthesia, McMaster University, 1280 Main Street West, HSC-2U Hamilton, Ontario, L8S 4K1, Canada; 3 Clinical Epidemiology and BiostatisticsBiostatistics Unit, FORSC 3rd Floor H325 50 Charlton Avenue East Hamilton, Ontario, L8N 4A6, Canada; 4 Department of Anesthesia and Pain Medicine (D-149), McMaster University St Joseph’s Hospital, 50 Charlton Avenue East Hamilton, Ontario, L8N 4A6, Canada

**Keywords:** Lumbar radicular pain, Pulse radio frequency, Dorsal root ganglion, Radiofrequency of DRG

## Abstract

**Background:**

Chronic lumbar radicular pain can be described as neuropathic pain along the distribution of a particular nerve root. The dorsal root ganglion has been implicated in its pathogenesis by giving rise to abnormal impulse generation as a result of irritation, direct compression and sensitization. Chronic lumbar radicular pain is commonly treated with medications, physiotherapy and epidural steroid injections. Epidural steroid injections are associated with several common and rarer side effects such as spinal cord infarction and death. It is essential and advantageous to look for alternate interventions which could be effective with fewer side effects.

Pulse radio frequency is a relatively new technique and is less destructive then conventional radiofrequency. Safety and effectiveness of pulse radio frequency in neuropathic pain has been demonstrated in animal and humans studies. Although its effects on dorsal root ganglion have been studied in animals there is only one randomized control trial in literature demonstrating its effectiveness in cervical radicular pain and none in lumbar radicular pain. Our primary objective is to study the feasibility of a larger trial in terms of recruitment and methodology. Secondary objectives are to compare the treatment effects and side effects.

**Methods/designs:**

This is a single-center, parallel, placebo-controlled, triple-blinded (patients, care-givers, and outcome assessors), randomized control trial. Participants will have a history of chronic lumbar radicular pain for at least 4 months in duration. Once randomized, all patients will have an intervention involving fluoroscopy guided needle placement to appropriate dorsal root ganglion. After test stimulation in both groups; the study group will have a pulse radio frequency treatment at 42°C for 120 s to the dorsal root ganglion, with the control group having only low intensity test stimulation for the same duration. Primary outcome is to recruit at least four patients every month with 80% of eligible patients being recruited. Secondary outcomes would be to assess success of intervention through change in the visual analogue scale measured at 4 weeks post intervention and side effects. Allocation to each group will be a 1:1 ratio with allocation block sizes of 2, 4, and 6.

**Trial registration:**

ClinicalTrials.gov NCT01117870

## Background

Chronic lumbar radicular pain (CLR) refers to symptoms of neuropathic pain in the territory of the affected lumbar nerve root. More precisely, the pathology in this condition affects a particular nerve root after it exists from the spinal canal, and before it becomes a part of the somatic nerve. The quality of this pain is usually sharp, lancinating, or burning. Clear distinction must be made between radicular pain (as described above) and radiculopathy. Radiculopathy refers to objective loss of sensory and/or motor function as a result of conduction block and leads to features of numbness, motor loss, wasting, weakness, and loss of reflexes [1]. The patho-physiology of radicular pain is complex, with mechanical [2] inflammatory [3,4], and immunological factors playing a role. The dorsal root ganglion (DRG) has been implicated in its pathogenesis by giving rise to sustained impulse transmission as a result of direct compression or as a site of hyper-excited structure. Prolonged compression presumably accompanied by pathological changes in the nerve root or DRG causes radicular pain to develop [5]. The majority of patients with acute radicular pain due to a symptomatic herniated disc improve with conservative or no treatment and have minimal pain by 3 months. However a minority (less than 5%) go on to suffer from significant chronic pain [6]. Radicular pain is mostly treated with medications, physiotherapy, and epidural steroid injections (ESI). ESI, although effective in reducing short-term pain in most patients, is associated with side effects such as headaches, flushing, water retention, metabolic and endocrine changes like glucose intolerance, and adrenal suppression [7,8]. They are also known to be associated with potentially serious side effects such as spinal cord infarction and death secondary to intra-arterial injection of particulate steroid preparations [9]. It is clinically imperative and beneficial to look for alternate interventions which could be effective with fewer and/or lesser side effects.

**Figure 1:**
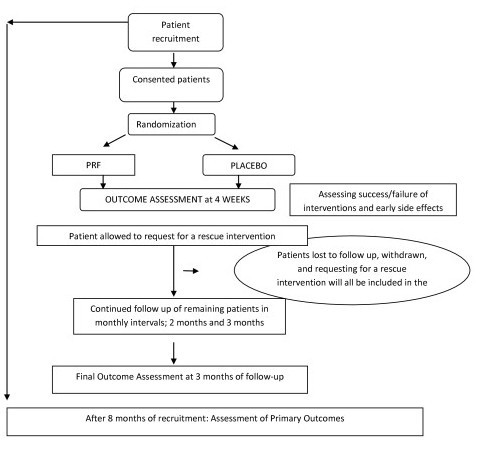
Randomized, placebo/sham controlled, double blinded trial study flowchart.

Pulsed radio frequency (PRF) is a relatively new technique derived from a well established and proven intervention called thermal radiofrequency (RF) denervation (ablation) [10]. Conventional RF treatment uses a constant output of high-frequency electric current, producing controllable tissue destruction surrounding the tip of the treatment cannula. Because of the thermal lesion (up to 80°C), it is associated with non-selective damage to neural elements and is known to cause deafferentation pain, which could be more severe than the original pain [11]. The comparisons of PRF and continuous RF are given in Table 1. Effectiveness of PRF in neuropathic pain and its safety have been clearly demonstrated in animal studies and as well as studies done on humans [12]. Various studies have demonstrated its possible mechanisms of action, including non-thermal effects on DRG, selective inhibition of pain carrying fibers, and activation of C-fos. At least two recent and well-conducted studies [13,14] and one case series [15] are an indication of its effectiveness specific to lumbar radicular pain. However, there has been only one randomized controlled trial (RCT) [16] in literature demonstrating its effectiveness in cervical radicular pain and none in lumbar radicular pain. The development and present use of PRF, as an interventional tool in pain management, has been pictorially compared to a ‘train running in reverse’ (as a refinement - came out to be widely used and successful but never bench tested) by Cohen and Zundert [17]. Unfortunately the use and advocation of PRF as a novel and successful treatment for many painful conditions is in practice without having the necessary scientific evidence, especially in the form of well controlled RCTs [18,19]. It has made many clinicians to question its scientific basis and evidence in terms of specific disease-based application [12,18-20]. Rapidly changing theories regarding its mechanism of action have not helped matters. In various evidence classifications schemes across the world, well-controlled RCTs are seen as the gold standard evidence of the effects of treatments. To receive a class I and grade A evidence classification, an intervention has to have at least one positive, adequately powered and well-conducted RCT or, preferably, a number of RCTs that consistently show the benefit of therapy [21]. We conducted a thorough literature search in MEDLINE (pubmed), EMBASE, and Cochrane database - with the terms ‘pulsed radiofrequency’, ‘pulse radiofrequency’, ‘pulse radiofrequency of DRG’, ‘dorsal root ganglion treatment with pulsed radiofrequency’, and ‘PRF-DRG’. No limits were applied for the period of search. Up to 2010, we obtained 368 citations. Abstracts pertaining to electrical field research and treatment for non-painful conditions were omitted. Potentially relevant articles were screened and articles pertaining only to ‘PRF treatment in pain’ were selected giving rise to 85 articles. Only abstract submissions or conference proceedings were not included. We observed that most of the evidence regarding the use of PRF in chronic pain conditions consists of retrospective and prospective case series. Only a few RCTs concerning the use of PRF was found in our literature search; Kroll *et al.*[22] and Tekin *et al.*[23] on facet joint pain-PRF of medial branch, Erdine *et al.*[24] on PRF of gasserian ganglion for trigeminal neuralgia, Van Zundert *et al.*[16] and Simopoulos (pilot study) [25] for radicular pain. The study by Van Zundert *et al.* is the only RCT concerning the application of PRF-DRG for cervical radicular pain. Its main limitation was inadequate number of subjects. There has not been any RCT involving PRF-DRG for lumbosacral radicular pain.

**Table 1:** **Comparison of continuous*vs.*pulse RF**

	**Continuous RF**	**Pulsed RF**
First used	Shealy 1975	Sluijter and others 1998
Application	Continuous RF energy for 90 s	RF energy in pulses of 20 ms with wash-out period of 480 ms
Needle tip	Parallel and by the side of target	Perpendicular, pointing at the target
Tissue temperature achieved	Up to 80°C	Up to 42°C
Proposed mechanism of action	Non-selective thermal destruction	Neurobiological - by strong electrical fields
Side effects	Deafferentation syndrome	No side effects observed so far
Duration of effect	Potentially long lasting (months)	? Relatively short duration
Use on peripheral nerves	Cannot be used -contraindicated	Has been effectively used in peripheral mono-neuropathies

The resulting articles were then studied and grouped into categories: A, basic science and animal experiments-demonstrating its efficacy, safety and mechanisms of action (summarized in Table 2); B, systematic reviews (considered below); C, application of PRF for spinal pain conditions-headache, back pain and radicular pain, and application of PRF to DRG (summarized in Table 3); D, application of PRF in various other painful clinical conditions (not considered relevant for this study).

**Table 2:** PRF in animal experiments demonstrating its effect and safety

**Authors**	**Study information**	**Results**	**Observations**
Tun *et al.*[26]	Histopathology and electron microscopy of rat brain tissue after RF and PRF	Reversible histological changes with PRF, while cellular necrosis seen with Cont RF	PRF is non-destructive and safe than RF
Cahana *et al.*[27]	Acute effects of PRF and CRF on impulse propagation and synaptic transmission in rat hippocampus	Effects of PRF less destructive and reversible. At more than 2000 μm, both RF and PRF did not affect the cellular morphology, at 1000 μm only CRF and not PRF destroyed the neuronal architecture	PRF is safer when compared to CRF; also established the clinical effects with differing distances from the RF probe
Higuchi *et al.*[28]	Rat DRG exposed to PRF and CRF	Only PRF led to increased C-Fos expression from the superficial laminae of the dorsal horn within 3 h after treatment	Action of PRF was demonstrated by C-Fos expression, which is speculated to be a mode of action of PRF
Erdine *et al.*[29]	Rat DRG exposed to CRF and PRF	Mitochondrial degeneration and loss of nuclear membrane with CRF and only cellular edema with PRF (reversible change)	Safety of PRF over CRF on DRG was demonstrated
Van Zundert *et al.*[30]	Rat DRG CRF and PRF applied at 67°	Demonstration of late trans-synaptic activity as C-Fos seen even after 7 days in both RF and PRF	Late cellular activity observed by PRF; indicates long-term changes effected by PRF
Podhajsky *et al.*[31]	CRF and PRF at 2° on rat DRG	Only endoneurial edema and collagen deposition; no irreversible changes seen	PRF depends on non destructive effects, and temperature of 42° and below are not associated with neural destruction
Hamman *et al.*[32]	PRF applied to axotomised DRG and sciatic nerve	Increased ATP -three positive cells with PRF of axotomised DRG and not in sciatic nerve	Selective action of PRF on Aδ and C fibers, sparing the more larger fibers
Hagiwara *et al.*[33]	PRF on rats with adjuvant induced hyperalgesia	PRF at 37° and 42°-successful in treating hyperalgesia, when compared to CRF and sham interventions The same effect was blocked by α-adrenoceptor blocker Yohimbine	The analgesic effect of PRF may involve descending adrenergic and serotenergic systems
Aksu *et al.*[34]	Rat model - induction of neuropathic pain by sciatic nerve ligation and PRF to DRG	PRF successful in decreasing the hyperalgesia associated with neuropathic pain	Efficacy of PRF in neuropathic pain
Heavner *et al.*[35]	Coagulation of egg white patterns with CRF and PRF applied at various temperatures	At 42°- no coagulation observed with PRF, and pattern just observed at 60°, with CRF coagulation observed even at 42°	Safety of PRF when applied at 42°
Vatansaver *et al.*[36]	Neurothermal effects of PRF and CRF - studied in sciatic nerve of rats, with lesions applied at 400°C, 420°C, and 800°C	No neurological deficits at temperatures less than 800°C; however at 400°C, PRF was less damaging than CRF	Relatively safety of PRF further established at less than 42°C

**Table 3:** Studies demonstrating the effects of PRF on spinal pain conditions

**Studies**	**Patients and treatments**	**Results**	**Observations**
Van Zundert *et al.*[37]	18 patients with cervical headache and cervico-brachialgia; PRF-DRG	13/18 patients >50% pain relief at 8 weeks, at 1 year 6 patients had continuing pain relief; no complications reported	First documented evidence of PRF treatment in cervical syndromes
Van Zundert *et al.*[16] P, R, DB, RCT, sham controlled	23 patients with Cervico brachial pain; 11 patients had PRF-DRG and 12 had Sham	3 months - 82% patients in the PRF-DRG group and 25–33% in the Sham group had successful results (*P* = 0.02–0.03)	PRF-DRG may provide pain relief in patients with cervico-brachial pain
Tsou *et al.*[38] Retrospective	127 patients; group A - back pain without lower limb pain, group B - back pain with lower limb pain	Successful treatment shown; At 3 months: Group A - 27/45 and Group B - 37/78 patients At 1 year: Group A - 20–45 patients and Group B - 34/74 patients	Pulsed radiofrequency applied at the L-2 DRG is safe and effective for treating for chronic low-back pain
Kroll *et al.*[22] Prospective, DB, randomised	50 patients treated with CRF or PRF of lumbar facets, and assessed with VAS, ODI -measured at baseline and 3 months	No difference in the two groups, however over time the CRF patients showed better scores than PRF	Effects of PRF may be limited by time when compared to CRF
Simopoulous *et al.*[25] Pilot - prospective RCT	26 patients with lumbosacral radicular pain grouped to PRF-DRG or PRF-DRG followed by CRF-DRG	At 2 months 70% of PRF showed significant reduction of pain scores compared to 83% in CRF after PRF, no statistical difference	PRF-DRG appears to be a good treatment without side effects for lumbosacral radicular pain
Lindner *et al.*[39] Retrospective study	48 patients with positive diagnostic blockade of lumbar medial branch, had PRF	21/29 patients with no previous surgery and 5/19 patients with previous surgery showed successful pain relief at 4 months, significant difference in PRF efficacy in between groups (*P* = 0.0028)	PRF of lumbar medial branch for facetogenic pain is safe and works well in patients who have not had back surgeries
Texiera *et al.*[40] Prospective, case series	8 patients with discography confirmed discogenic pain - intradiscal PRF	Significant drop in NRS scores at 3 months, 4 patients were reportedly pain free after 12 months	Intradiscal PRF merits a controlled prospective study
Chao *et al.*[15]	154 patients with cervical (*n* = 49), lumbar (*n* = 105) radicular pain due to herniated disc and FBSS	At 3 months 27/49 in cervical and 52/105 in lumbar patients had pain relief >50%	Application of PRF is a safe and useful intervention for cervical and lumbar radicular pain
Texiera *et al.*[14] Retrospective study	13 patients with lumbosacral radicular pain due to herniated disc had PRF-DRG	Significant pain reduction (*P* = 0.01), was found in 11 patients from 4 weeks lasting up to 15 months, only 1 patient had a small area of low sensation at L3 area in the last follow-up	PRF may potentially be a viable alternative for epidural steroid injections in the treatment of radicular pain
Shabat *et al.*[41] Retrospective	28 patients with chronic neuropathic pain of spinal origin had PRF-DRG	19 patients had successful pain relied lasting for an year, with no reported complication	PRF is a safe and an effective procedure for patients who suffer from chronic neuropathic pain from spinal origin
Tekin *et al.*[23] Prospective RCT	60 patients grouped with clinical diagnosis of facet joint pain - grouped into LA, PRF, and CRF groups	Pain relief in PRF and CRF better, however in the follow-up period the relief was not sustained in the PRF group	Pain relief with PRF is comparable to CRF, but the duration of effect is shorter
Mikeladze *et al.*[42] Retrospective study	114 patients with cervical and lumbar pain, responsive to diagnostic medial branch block-PRF	68 patients had significant pain relief lasting at least 4 months	PRF of medial branch is a successful intervention in selected patients with no complications

### Systematic reviews

Because of the lack of sufficient RCTs systematic reviews were considered. Although there have been at least eight literature reviews on the use of radio frequency in chronic pain, only six of them have included the use of PRF. Geurts *et al.*’s [43] review considered the use of conventional RF only, in spinal pain. Neimisto *et al.*[44] conducted a systematic review in the frame work of Cochrane collaboration; even this review was focused only on conventional RF. Zundert *et al.*[45] could not make a detailed review of PRF, as the available evidence was limited at that time. Malik and Benzon [46] included studies concerning application of both RF and PRF specifically to DRG in their recent review. They included one RCT by Van Zundert on cervical DRG and three retrospective studies and few case series and reports. However their opinion on use of PRF of DRG was limited by the small number of studies. In an earlier article, Malik and Benzon [47] made a critical review on the efficacy of PRF. They stated the need for further RCTs in PRF in order to help the practicing pain physician in their use of PRF for many chronic pain conditions. Boxem *et al.*[48] reviewed RF and PRF with the view of assimilating the present available evidence for their use in various chronic pain conditions. The evidence was supposedly similar for both RF and PRF in cervical radicular pain but they opined that PRF should be preferred as it is associated with lesser side effects. Bryd and Mackey [49] performed an excellent review of PRF including its history and its applications in various pain conditions. Apart from recognizing that evidence in the form of RCTs are lacking, they stated that the emergence of PRF technology represents a promising step toward treating complicated pain conditions. As the evidence in support of PRF accumulates, it is likely that its potential to be applied more broadly will also increase. Cahana *et al.*[12] did a literature review solely on PRF. Their literature search revealed several prospective and retrospective studies along with many case reports. Fifty-eight reports were considered in the final evaluation. They observed that PRF treatment elicits a genuine neurobiological phenomenon altering the pain signal; however the mechanism of action is not completely elucidated. There are no major or significant side effects related to PRF reported to date. The views and positions of many senior and experienced clinicians on PRF are conflicting. Some are convinced by the scientific evidence that PRF is genuinely effective [10,17,50] for neuropathic pain. However the consensus view points towards inadequate evidence as compared to other evidence-based treatments. Most are of the view that there needs to be further studies (RCTs), which clearly establishes its role in specific target population [10,18-20,48,51].

In this ‘proof of concept’ study we would like to answer the question, ‘whether PRF, when compared to placebo, is effective in reducing pain in chronic lumbar radiculopathy (CLR)’? With this rationale and purpose, a feasibility study is being done to assess whether a larger scale clinical study with the same methodology can be done. This pilot study would also help in assessing the actual number of patients to be involved in the larger clinical study to give a statistically significant difference. With positive outcomes (treatment success), we could also determine the length of follow-up necessary in a full-scale clinical study in order to study the duration of effects.

### Primary research question

Is it feasible to do a larger scale clinical study to study the effectiveness of ‘PRF treatment of DRG as compared to placebo’ in CLR? Feasibility (outcome) parameters studied: (1) recruitment rate; (2) percentage of patients who go on to complete the full study after enrolling as participants.

### Secondary research questions

1. Is pulsed radiofrequency of DRG effective for pain relief - measured as a decrease in VAS, measured at 4 weeks?

2. Is application of PRF associated with any short-term (at 4 weeks) or long-term persisting (beyond 3 months) side effects?

3. Is there any improvement in Oswestry Disability Index (ODI) in patients of CLR after PRF; measured at 4 weeks, 2 months, and 3 months?

4. Is there any decrease in analgesic (medications) use in patients with CLR after application of PRF; measured at 1 week, 4 weeks, 2 months, and 3 months?

## Methods/design

### Methodology and techniques

Patients would be screened at St Joseph Hospital, Hamilton, Canada, at their-East End Pain Clinic for CLR of at least 4 months’ duration. Based on the history and clinical examination, a diagnosis of CLR involving one or more spinal segments is made and noted. The patient is considered eligible for the study if a CT or MRI of lumbar spine done within the last 4 months demonstrates pathology that is concordant with the patient’s clinical symptoms. The spinal level(s) targeted for treatment will be based on clinical findings, for example if a patient exhibits signs and symptoms of right L4 radiculopathy, and the MRI demonstrates right paracentral disc herniations at both L4-5 and L5-S1, only the L4 DRG will be targeted. Patients fulfilling the inclusion criteria will be fully explained about the nature of study, interventions involved and the possible complications after which an informed consent will be taken. Enrolled patients would be randomized according to the method described and included in the study according to previously randomized order. He/she will also meet the assessor (blind to intervention), who would note down the baseline parameters of the patient and also collect a baseline ODI score along with noting down other parameters. A patient having bilateral radiculopathy will be treated for his most affected side in the trial. A patient having CLR of more than one segment on one side shall be treated for all the involved segments with the same technique and counted as a single procedure or intervention for the study. After checking for the informed consent and other safety checklist, patient will only be revealed as belonging to the study (not mentioning whether it is a placebo/actual treatment). All operating room (OR) personnel, including the physician performing the intervention and the patient, shall be blind to the randomization and treatment. The grouping code, hence the actual treatment will only be known by the person operating the RF machine. Only the RF technician shall be in view of the working details of the RF machine and the noise of the machine is cut by playing out a music or song. This ensures blinding of all involved, except the RF technician. All interventions will be done as day-care procedures in the OR. Patients would continue to use their medications as before. If the pain relief obtained necessitates decrease in the usage of medications that shall be recorded. Similarly unsatisfactory pain relief obtained necessitating increase in the dose or change of medications shall be recorded. The other parameters to be used for statistical analysis are collected as mentioned under data analysis. The patient will have an established IV access. He would be put in prone position. The involved area of back would be made sterile using chlorohexidine 2% and draped. The patient would be connected to continuous monitoring of 3 lead ECG, NIBP, and pulse oximetry. Sedation, if used will be minimal (Grade I or II) to obtain the necessary response of the patient. Under fluoroscopy guidance particular spinal segment(s) affected is (are) identified and confirmed. LA using 2% lidocaine is infiltrated to the skin at entry site. For both groups a RF needle (Bayliss: 22-G needle, 5-mm curved active tip and 10 cm) is used. With an appropriate fluoroscopy view the needle is inserted to the target location in both the groups.

Target location: the DRG, which is an enlargement formed by the dorsal nerve root just proximal to its junction with the spinal nerve, lies within the dural sleeve and occupies the upper, medial part of the intervertebral foramen [46]. It is confirmed with an anteroposterior fluoroscopy view in which it is advanced, if required, until the tip is located one-third to halfway into the pedicle column.

Target confirmation [46]: appropriate fluoroscopic placement of needle near DRG at the lumbar area is noted; on anteroposterior X-ray projection, the DRG is described to lie immediately behind the lateral aspect of the facet column at all spinal levels and on lateral X-ray projection, it is localized to the dorsocranial quadrant of the intervertebral foramen (IVF). Proximity of the needle to the DRG is determined by appropriate sensory stimulation with 50 Hz, at more than 0.4 V (avoids intra-ganglionic placement), and less than or equal to 0.6 V; motor stimulation at 2 Hz with threshold 1.5-2 times greater than sensory threshold to avoid placement near the anterior nerve root. A radiculogram done also confirms the appropriate placement and helps recognize intradural placement of the needle. Both the groups will have their respective DRG stimulated for sensory confirmation. Only lidocaine 1% 0.5 mL shall be given in both groups before carrying out the treatment.

Treatment: once positioned the physician shall indicate to the RF technician as ‘treatment’ only, at which time either PRF or placebo (only continuing sensory stimulation at a low frequency of 0.2 V is applied) without revealing. Application of intervention: in Group A, PRF; PRF would be applied for 120 s at 42°C. Group B, placebo; the needle would be continuously stimulated at a low voltage to give a sensation of PRF application and also to obtain the necessary noise to blind the patients. In both groups the same procedure is done at all the involved spinal segmental levels. After the procedure the patient is shifted to recovery area to be monitored, observed and managed for any side effects. The observer, blind to the interventions, will record the pain scores and also check for side effects observed before the patient is discharged home.

Blinding and bias control: in our study, the patient, the treating physician, and the assessor for the actual intervention are all blinded. The randomization code, hence the actual treatment, will only be known and administered by the person operating the RF machine. The randomization will be concealed in sealed envelopes. Blinding of patients will be achieved by randomization, use of similar technique and fluoroscopy, stimulating the DRG in both groups, and continuing non-PRF stimulation in placebo group, use of audio to mask the treatment sound. Blinding of the treating physician and assessor will be achieved by not knowing the randomization order and hence not aware of the actual treatment.

### Planned inclusion and exclusion criteria

Suitable patients older than 18 years, suffering from CLR of at least 4 months or more and with concordant findings on either MRI or CT are included. Included patients must also have a VAS score of at least 6/10 (at presentation) and must be ready to provide informed patient consent for participating in the study as a blind subject.

Exclusion criteria would include any patient having an absolute contraindication to neuraxial injection in the form coagulation disturbance anticoagulant therapy, bleeding disorder, or infection at the site of injection; patients with anatomical deformity or derangement, either congenital or acquired such as extreme scoliosis, previous implant or instrumentation, making it difficult to access the foramen as evidenced by MRI, CT, or plain X-rays; patients with cancer to account for their symptoms; patients with known significant psychiatric history; patients unable to communicate effectively in English; patients with allergy to local anesthetics or radiographic dye; patients with a history of acute neurological weakness or neurodeficit in the affected limb in terms of measurable motor weakness or abnormal reflexes.

### Duration of treatment and follow-up

Duration of treatment period will involve approximately 30 to 60 min of actual intervention done in the OR. The duration of complete follow-up will be for 3 months after the intervention with the following interval follow-up assessments: (1) 30 min post-procedure in recovery, (2) 24 h after - by a phone call (only VAS and side effects); (3) at 1 week post-procedure visit to the assessor (VAS, ODI, medications, side effects); (4) at 4 weeks post-procedure visit to the observer for assessment for success (VAS, ODI, medications, side effects); (5) at 2 months post-procedure-visit to observer (VAS, ODI, medications, side effects); (6) at 3 months post-procedure visit to the observer - last follow-up visit (VAS, ODI, medications, side effects).

### Primary and secondary outcome measures

#### Primary

1. Recruitment rate: percentage of suitable patients fulfilling the inclusion–exclusion criteria, recruited for the actual study after informed consent. The final assessment will be after the complete recruitment, at which time all the expected number of subjects (*n* = 32) must have been enrolled. Criteria for success would be as expected recruitment of at least four patients per month after fulfilling the selection criteria and with full informed consent with at least 80% of eligible patients fulfilling the selection criteria being recruited.

2. Percentage of patients continuing to be participants for the whole study.

Participants retain the right to withdraw from the study at any point. However all participants after intervention shall be included in the final analysis, on intention to treat principle. Any participant requesting for a withdrawal shall be given the option of continuing medical care or epidural or transforaminal ESI at the next available opportunity (present standard of care).

#### Secondary

1. Effectiveness of PRF treatment of DRG, when compared to placebo, as a treatment in patients with CLR: outcome measured as decrease in VAS scores (0 to 10) from baseline measured at recruitment. For definition of success we would consider at least 50% decrease in VAS scores assessed at 4 weeks.

2. Assessment of short-term side effects: percentage of patients having side effects after PRF treatment assessed at 1 week, compared with the placebo group. Assessment of persisting side effects: percentage of patients having side effects after PRF treatment, beyond 1 week, compared with placebo group. Short-term side effects (within 1 week – recorded at the first visit):

Nausea

Headache

Transient increase in pain

Fever (temperature in°C)

Transient paresis/paraesthesia

Dysesthesia

Long-term side effects (persisting side effects assessed at visits 1 week and at 6 weeks):

Infection at the site

Meningitis

Epidural abscess

Hyposensibility

Paralysis/paresis

Dysesthesiae

Any other

3. Improvement in ODI: success defined as at least 50% improvement (or decrease) in ODI measured at 4 weeks, compared with placebo group.

4. Decrease in the analgesic medications used: percentage of patients with increase or decrease in their medication use (either in dose, frequency), compared with placebo group.

### Rationale behind the assessment of interventions for success at 4 weeks

1. Four weeks can be considered as the optimum time at which the therapeutic effect of PRF would be at its maximum.

2. There is a theoretical possibility of local anesthetic action in either group, which can be eliminated at 4 weeks.

3. Even if there has been a placebo effect it is important to know whether it is sustained at 4 weeks, in comparison to the actual treatment effect.

4. To allow appropriate time for a decrease in ODI (functional assessment) to happen as a result of decreasing pain.

### Trial design, outline, and flow chart

#### Sample size, recruitment rate

Sample size has been determined based on feasibility considerations. Total duration of recruitment planned is 8 (clinically active) months. The proposed target is recruitment for 8 months, with an expectation of at least four patients every month, with a total of 32 patients for the study. Potential to recruit patients, as four patients every month has been calculated based on the transforaminal steroid injections (TFESI) performed in the last 3 to 6 months and booked for the next 3 to 6 months (calculated as monthly) at St Joseph’s Healthcare; data obtained from St Joseph’s Healthcare, Department of Anesthesia and Pain. TFESI represents a well-accepted, presently performed intervention for patients of CLR and most patients suitable for that are potential recruits for this study.

#### Randomization

Patients will be randomly allocated to each group using a 1:1 ratio. The allocation will be done in blocks using block sizes of 2, 4, and 6.

#### Statistical tests

The characteristics of the trial participants will be described using mean (standard deviation) or counts as appropriate. We will use a flow diagram to summarize the flow of patients in the trial. Feasibility outcomes will be reported as counts (proportions).

## Discussion

The results of this pilot study would primarily give us information about the patient recruitment and study methodology. This is being done as there are no previous studies (RCTs) to indicate the successful effects of PRF in CLR. The initial study proposal was to conduct an RCT comparing PRF-DRG with TFESI, which have proven to be more successful than midline ESIs for lumbar radiculopathy [52], and are probably the most effective intervention available. However our literature search showed that so far, there were no RCTs involving sufficient participant numbers to evaluate the efficacy of PRF. The only RCT by Van Zundert *et al.*[16] on cervical radicular pain had only 19 participants in either group. Keeping this in mind we devised this proof of concept study to evaluate the efficacy of PRF as compared to placebo.

It is very challenging to devise and conduct a placebo controlled trial in interventional pain medicine. Because of the nature of the problem, which involves significant suffering for a long time, patients may be unwilling to participate as blind participants. CLR patients would have had a trial of most medications and simple interventions before they go on to have complex spinal interventions, such as PRF-DRG. It may also be difficult to have such patients to be under long periods of follow-up after a blind intervention, especially if it is not of benefit to the patient. This trial would be able to tell us whether we were able to recruit patients as expected for the trial and also whether they continue to be participants of the study for the whole duration. The other important and novel aspect of this study which merits attention is the technique of patient blinding during the procedure. Although we will not be formally evaluating the blinding process, it will be interesting to know if we faced significant challenges and if such a methodology needs to be appropriately modified for a future trial. The study would also assess the outcomes of pain relief from PRF compared to placebo. Apart from telling us if PRF is successful, the study would also indicate the difference in pain relief in terms of absolute reduction in VAS scores. This is necessary to assess the number of study participants for a future trial. The assessment of side effects is included to understand if there are major or minor side effects and risks which need to be better studied and informed to the patients.

## Trial status

We are presently recruiting study participants. The trial is registered on clinicaltrials.gov with the unique identifier NCT01117870. We only require two more participants to complete the recruitment process. We have also completed interventions on 28/32 patients.

## Abbreviations

CLR: Chronic lumbar radiculopathy; DRG: Dorsal root ganglion; ESI: Epidural steroid injection; PRF: Pulse radiofrequency; RF: Radiofrequency; C-Fos-Proto-oncogene: Protein coded by FOS gene; RCT: Randomized control trial; VAS: Visual analogue scale; ODI: Oswestry Disability Index; OR: Operating room; IVF: Inter-vertebral foramen; TFESI: Transforaminal epidural steroid injection; LA: Local anesthetic; CRPS: Complex regional pain syndrome; TENS: Trans-cutaneous electrical nerve stimulation.

## Competing interests

No financial or non-financial competing interest exists concerning any authors.

## Authors’ contributions

HS: Lead investigator, corresponding author of the manuscript, and treating physician for the study patients. PC: Local primary investigator and treating physician involved in patient recruitment, treatment, and follow-up of patients. JMC: Co-investigator and treating physician involved in patient recruitment, treatment, and follow-up of patients. JP: Co-investigator involved in methodology planning, original protocol drafting, and supervision. LT: Co-investigator, statistical assistance, and supervision for the study. All authors read and approved the final manuscript.
